# Research note: The effect of passionflower supplementation on feather pecking in laying hens

**DOI:** 10.1016/j.psj.2025.105102

**Published:** 2025-03-26

**Authors:** Elizabeth Brass, Jack O'Sullivan, Helen Gray

**Affiliations:** School of Natural and Environmental Sciences, Newcastle University, UK, NE1 7RU

**Keywords:** Feather pecking, Laying hen, Welfare, Botanicals, Aggression

## Abstract

Feather pecking is a significant issue in non-caged poultry welfare that results in the removal or damage of the feather of a hen. The most common forms are classified into gentle feather pecking and severe feather pecking which, if undeterred, can develop into cannibalism. This case study explored one aspect of the prevention of feather pecking, investigating if the feed additive Gallicalm, containing Passionflower, reduced feather pecking behavior in a free-range flock.

Video footage over 6 weeks was analysed for feather pecking incidence in 2-week phases; Pre-Treatment, Treatment and Post-Treatment. Standard commercial rations were fed in the Pre-Treatment and Post-Treatment phases, with the Treatment phase receiving the standard commercial ration plus 1 kg per ton of Gallicalm. Feather scores were completed using the AssureWel method at the end of each phase, with production data collected through an online flock management tool. A total of 373 minutes of footage from 18 days was analysed for pecking behavior.

Supplementation resulted in reduced number of severe feather pecks in the Pre-Treatment phase to the Treatment phase. Gentle pecking failed to decrease significantly during Gallicalm Treatment but increased in the post-Treatment phase. Aggressive, stereotypical and beak pecking were rare in all experimental phases. Feather scores deteriorated between the Pre-Treatment and Treatment phase but plateaued between the Treatment and Post-Treatment phase.

This case study provides the first evidence of passionflower-containing supplements reducing feather pecking in laying hens. Given the billions of laying hens kept globally and the extensive welfare and economic issues associated with feather pecking, we advocate for further study to build on our initial findings.

## Introduction

Feather Pecking is an abnormal behavior where one bird pecks at the feathers of another bird, resulting in feather damage or even the feather being plucked. As one of the largest threats to non-caged laying hen welfare, prevalence rates are high (Nicol et al., 2013).

Gentle feather pecking (GFP) and severe feather pecking (SFP) are the two common forms of feather pecking. GFP occurs when a bird gently pecks the feathers of another, with the recipient remaining undisturbed. Conversely, SFP entails forceful pecks that pluck feathers out, resulting in the victim moving away (Rodenburg et al., 2013). Considered a gateway behavior, GFP frequently escalates into SFP and cannibalism (Rodenburg et al., 2013). Such behavior not only compromises bird welfare but can negatively impact economic profitability (Nicol et al., 2013).

Feather pecking is a multifactorial behavior, influenced by stress management, nutrition and genetics (Rodenburg et al., 2013). Current prevention strategies focus on stress reduction and enrichment, with dietary interventions such as increasing insoluble fiber, playing a key role in reducing feather pecking (Rodenburg et al., 2013). Insoluble fiber dilutes the diet, extending the feeding time and promotes satiety, helping to mitigate feather pecking.

In other farmed species, vice behavior, such as tail biting in pigs, are becoming more commonly controlled by additives infused with botanicals. Recent research with botanicals such as passionflower *(Passiflora incarnat*a*)* have shown promise in reducing stress-related behaviors (Torre et al., 2022), though little research exists examining botanicals for feather pecking control. Although not tested on commercial poultry, reduced aggression, fewer injuries and lower cortisol levels were found in Japanese Quail when supplemented with passionflower (Torre et al., 2022). Therefore, this study aims to explore the potential of passionflower feed additives to alleviate an outbreak of feather pecking with the following hypothesis and predictions:

H0 The addition of passionflower in-feed using the product Gallicalm will have no effect on the hens during a feather pecking outbreak.

H1 The addition of passionflower in-feed using the product Gallicalm will reduce feather pecking and aggressive pecking, and feather coverage will be prevented from worsening during the Treatment phase.

## Materials and methods

This project was completed in line with the stipulated ethics from Newcastle University's Animal Welfare and Ethical Review Body (Ref 1076).

### Animals and housing

The study took place in Northwest England from January 7^th^ to February 21^st^ 2024, on a H & N Brown free-range flock using a Big Dutchman flat deck system. The litter and range were located on one side of the shed.

Observations were taken from 27 to 34 weeks of age on a flock of 11,947 chickens. Observations were split into 3 two-week phases: a control phase on a standard commercial diet; a Treatment phase on the experimental diet (commercial ration plus Gallicalm); a post-Treatment phase returning to the standard diet. A two-day acclimation period occurred between the control and Treatment phases due to the mixing of feed rations. Gallicalm was incorporated at the feed mill at 1 kg per tonne of finished feed, containing less than 750 g of Passionflower per kg.

To check for adverse effects of the product, birds were weighed weekly from day 13 of the trial, with fecal scores and bird dirtiness assessed from the beginning of the Treatment phase until the end of the trial. Fifty birds were randomly selected throughout the house for hand weighing using BW-2050 scales (Weltech, UK). Fecal and bird dirtiness scores were assessed using the AssureWel methods, scoring fifty samples across the house daily. Fecal samples were scored from 0 to 3, with 0 representing a firm, healthy dropping to 3 representing a watery, unhealthy dropping. The entire body was scored for bird dirtiness, except the legs and feet, using a scale of 0 to 2 where 0 signifies a clean bird and 2 indicates substantial dirtiness (AssureWel, 2024a).

### Behavioral scoring

Feather pecking was assessed across all phases using video recordings from DS-2CD2346G2-IU cameras (Hikvision, China) connected with a DS-7608NI-K2/8P recorder (Hikvision, China). Two cameras were installed above the litter area of the poultry house, positioned to capture overhead views of the birds. Footage was recorded continuously between 05:30 and 20:00 throughout the trial, aligning with the lighting schedule in the poultry house. One minute video clips were observed using BORIS software (https://boris.unito.it) to record the behaviors described in Table 1. Clips were taken from 18 days of footage (for a total of 373 minutes), split across 6 days per phase, avoiding days where birds were weighed or feather scored. The time of day for observations was not equally matched across phases, but each hour from 06:00-20:00 had at least two observations per experimental phase.

**Table 1:** Ethogram used for behavioral scoring using categories: gentle feather pecking, stereotyped feather pecking, severe feather pecking, aggressive pecking and beak pecking.

Behavior	Description
Gentle Feather Pecking	Classified as mild pecking at the feathers of another bird. A ‘pecker’ will use its beak to gently peck at the feathers of a receiver. The receiver usually ignores the pecking and the behaviour doesn't result in a feather removal. Directed to the back or tail, not the face or neck. Usually, an isolated peck but can be multiple pecks in a bout. Count each peck.
Stereotyped Feather Pecking	Classified as mild pecking at the feathers of another bird. A ‘pecker’ will use its beak to gently peck at the feathers of a receiver. The receiver usually ignores the pecking and doesn't result in a feather removal. Directed to the back or tail, not the face or neck. Usually, multiple pecks in a bout. A ‘bout’ is classified as more than one peck repetitively in quick succession (usually 1 peak per second). A bout ends when pecking has stopped for over 4 seconds or when pecks are directed at another body part. Count the number of bouts.
Severe Feather Pecking	A ‘pecker’ uses its beak to forcefully peck at the victim which may result in the removal or damage of a feather. The receiver usually responds to pecking by moving away or retaliating. Directed mostly towards the back, rump or tail. Count each peck.
Aggressive Pecking	Occurs when the ‘pecker’ raises her head and forcefully stabs her beak at another hen. Usually directed at the head but can be directed at the body. The receiver shows avoidance behavior by ducking or moving away. Count the total number of pecks.
Beak Pecking	A ‘pecker’ uses her beak to gently peck at the receiver's beak, face or neck. Can be an isolated peck, or more commonly multiple pecks in a bout. A ‘bout’ is classified as more than one peck repetitively in quick succession (usually 1 peak per second). A bout ends when pecking has stopped for over 4 seconds or when pecks are directed at another body part. Count each peck or the number of bouts.

To reduce bias in behavioral scoring, timestamps were removed from video frames, and file names were anonymized to prevent identification of the experimental phase via the date. Each video was reviewed four times, with each playback focusing on a quadrant of the screen (e.g., the top left of the screen during the first viewing). All observable instances of the behaviors were scored as counts.

### Feather scoring

Feather scoring followed the AssureWel method used commercially in the RSPCA assurance scheme (AssureWel, 2024b). Scoring was completed at 29, 31 and 33 weeks of age at the end of each phase. A sample of fifty birds were scored in two areas: the head/neck and the back/vent. A score of 0,1 or 2 was allocated: 0 demonstrates no damage or missing feathers, while 2 indicates the highest feather loss and damage (example photographs are available in the cited methodology).

A single researcher (LB) conducted the scoring, assisted by a stockperson unaware of study phase to minimize selection bias. Birds were selected at random throughout the entire shed. Scoring consistency was assessed by inter-rater reliability, with photographs of 10 % of the selected birds per session taken for independent scoring by a second researcher (HG) also blinded to the trial phase. Bird numbers for photography were chosen using a random number generator and unknown to the stockperson during selection.

### Production data

Flock-level production data was captured throughout the entirety of the trial using an online data system, BirdBox (https://www.faifarms.com/birdbox/) to ensure no negative impact occurred between trial phases. The data collected was daily production percentage, feed consumed per bird per day, water consumption per bird per day, bird weight and daily mortality.

### Statistical analysis

All statistical analyses and data visualization were conducted using R (v4.3.2) via R Studio (v2023.12.0). We used the BRMS package (v2.20.4) to run Bayesian linear models. BRMS uses Stan programming language and estimates model parameters using Hamiltonian Monte Carlo. Four Markov chains were run, each with a warm-up period of 2,500 iterations and 5,000 iterations used for sampling. Thinning was set to 1. Convergence was checked using the Gelman-Rubin statistic with convergence indicated by values close to 1 and less than 1.05. Plots were produced using the ggplot2 package (v3.4.4).

The model run for each behavior is shown by the equation below:

B∼Poisson(λ)

log(λ)=α+β·P+log(C)

Where the behavior (*B*) is Poisson distributed with rate λ. The rate is a function of an intercept (α) and the effect of experimental phase (β·P). Given that count data are directly impacted by the number of observations, we also included an offset variable (log(*C*)), which accounts for the different number of video observations watched per day.

Feather scores for both the head and back were analysed with the predictor of experimental phase using cumulative ordinal regression models, with a probit link function. Cumulative ordinal models estimate thresholds in place of traditional intercepts, as they assume a continuous latent variable underlying the ordinal categories of scores. Inter rater reliability was calculated using weighted Cohen's Kappa.

We had no specific hypotheses to test for the production data but calculated descriptive statistics to check for any adverse impacts of the supplement on production.

## Results and discussion

In total, across 373, one-minute observations, we scored 953 instances of severe feather pecking, 541 of gentle, 67 stereotypical pecks, 88 beak pecks, and 90 aggressive pecks.

Inter-rater reliability was assessed by a second annotator (JO) independently coding 10 % of the video (144 quadrants). Both annotators identified feather pecking in 57 quadrants, with Cohen's kappa indicating fair agreement (k = 0.33, SD = 0.43). In the remaining quadrants, 58 showed no feather pecking according to both annotators, while in 29 only one annotator identified the presence of feather pecking. These discrepancies primarily arose in densely packed frames with limited visibility of individual chickens, suggesting potential challenges in accurately classifying the behavior under those conditions.

While these findings demonstrate acceptable inter-rater reliability, using the current ethogram, some refinements may improve agreement on severity of pecking levels. Specifically, clearer distinctions between severity levels or the consolidation of similar behaviors into broader categories could improve consistency. It is important to note that all behavioral data analysed in this study were annotated by a single observer (LB), thus eliminating any potential impact of inter-observer inconsistencies on the results. This inter-rater reliability assessment serves as a valuable evaluation of the ethogram and methodology, informing future applications and refinements.

### Behavior

**Fig. 1:**
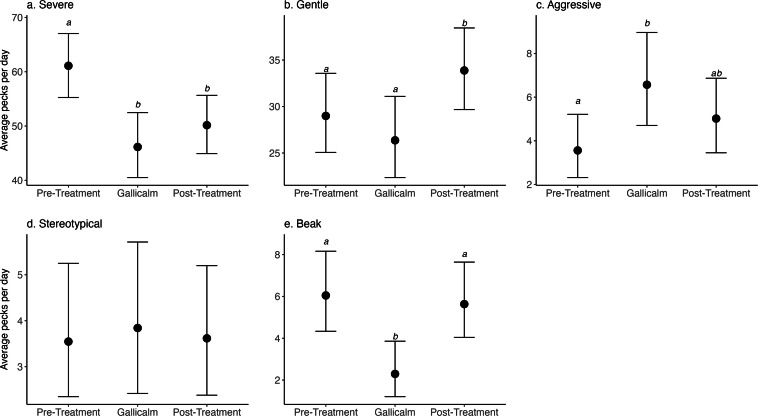
Model estimates (with 95 % highest density intervals) of the average number of pecks per day for each behavior category. Letters above points indicate significant differences between phases, where phases sharing a common letter are not significantly different, and phases with different letters are significantly different.

The most frequently observed behavior was severe feather pecking (Fig. 1a). The model estimated an average of 2.95 severe pecks per minute (95 % HDI: 2.67– 3.24) during Phase 1 observations. As the birds transitioned to Phase 2, we found severe pecks decreased by a multiplicative factor of 0.76 (95 % HDI: 0.64–0.88). This translates to 2.23 (95 % HDI: 1.95– 2.53) severe pecks per observation in Phase 2. The effect of reduction remained present during Phase 3, in which we observed a multiplicative effect of 0.82 (95 % HDI: 0.70 – 0.94), equating to 2.42 (95 % HDI: 2.16 – 2.69) severe pecks per observation. For both Phase 2 and Phase 3, therefore, the model estimates a decrease of ∼6 severe pecks every 10 minutes, compared with Phase 1. We saw no significant difference between Phase 2 and 3 (mean: 1.08, 95 % HDI: 0.92, 1.28).

Gentle feather pecking was the second most common behavior and was estimated at 1.40 (95 % HDI: 1.21, 1.62) events per minute in Phase 1. We found gentle pecking to be highest in Phase 3 (Fig. 1b), increasing from Phase 2 by a multiplicative factor of 1.29 (95 % HDI: 1.03, 1.60) to 1.64 events per minute.

Aggressive, stereotypical, and beak pecking were all rare and were estimated in Phase 1 to be 0.17 (95 % HDI: 0.11 – 0.24), 0.17 (95 % HDI: 0.11 – 0.25), and 0.29 (95 % HDI: 0.21- 0.39) pecks per minute, respectively. Experimental phase did not significantly impact stereotypical feather pecking. Beak pecking decreased in Phase 2 by a multiplicative factor of 0.40 (95 % HDI: 0.15 - 0.66) to 0.11 (95 % HDI: 0.06 – 0.18) instances per minute observation. Beak pecking then increased again in Phase 3 by a multiplicative factor of 2.49 (95 % HDI: 1.30 – 4.90). The rare nature of this behavior renders the effect on the number of pecks as minimal. There was a significant increase of aggressive pecking in Phase 2 by a multiplicative factor of 1.93 (95 % HDI: 0.99 - 2.97), but note that the lower HDI interval includes 1, signalling a very small effect size. We found no significant difference in aggressive pecking between Phase 2 and 3.

These behavior results support that the addition of Gallicalm reduced the severe and beak feather pecks, with short-term residual effects of the additive. For quantification of longer residual effects, further studies are needed. One explanation for the reduction is due to the presence of passionflower. While mode of action for the sedative effects of passionflower is uncertain, it is proposed that the compounds work on Gamma-aminobutyric acid (GABA; Elsas et al., 2010). GABA is a key inhibitory neurotransmitter linked closely with hyperactivity, irritability and obsessive behaviors such as SFP (Clemens Falker-Gieske et al., 2022). Modulation of GABA could therefore influence the neuronal excitatory state and reduce the incidence of obsessive-compulsive-like-behaviors.

Non-significant effects of Treatment seen in GFP and stereotypical pecking may indicate that different motivations and neurological pathways are used for reinforcement, which are unaffected by passionflower supplement. Aggressive pecking increased in the Treatment phase and did not return to Pre-Treatment levels throughout the remaining trials. This may be due to the development of the behavior in the flock over time, rather than influenced by the product, but further studies are required to evidence this.

### Feather scoring

Feather scores for both the back and head were significantly associated with the phase of the study. The effect of the Treatment phase shifted the estimated thresholds of head scores by 0.99 (95 % HDI: 0.51– 1.52) and by 1.22 (95 % HDI: 0.70 – 1.73) in the post-Treatment. This indicates the probability of a hen receiving a higher score (i.e. poorer feather cover) was increased for the Treatment and post-Treatment phases, compared with the control phase. The same relationship was seen for back scores at Treatment phase (estimate: 1.00, 95 % HDI: 0.51 - 1.47) and post-Treatment (estimate: 1.14; 95 % HDI: 0.61 – 1.60). Plumage scoring is well correlated with the rate of feather pecking (Schwarzer *et al.*, 2022). However, here we observe a worsening in feather scores during Treatment in contrast to the decrease we observed in severe pecks. This is not in-line with our prediction but may be due to the fact that severe feather pecking was not completely eradicated, meaning some pecking still resulted in feather loss.

Inter rater reliability for feather scores varied from moderate to substantial agreement. The head showed moderate agreement (κ = 0.48, *p* < 0.05). A comparison between phases of head scores showed the worst agreement was in the pre-Treatment phase (slight agreement; κ = 0.17), with moderate agreement in the post-Treatment (κ = 0.55) and Treatment phases (κ = 0.58). We believe the disagreement in scores is due to different contexts used for scoring. The second scorer (HG) scored from photographs due to biosecurity preservation, while the first scorer (LB) scored in person. Differences in lighting and bird presentation could explain the differences in scoring. The back showed substantial agreement (κ = 0.75, *p* < 0.01). A comparison between phases showed substantial agreement in post-Treatment (κ = 0.67) and Treatment (κ = 0.80) with perfect agreement in pre-Treatment (κ = 1).

### Production data

Descriptive statistics were performed on the performance data to check for adverse effects of the product. Egg production (percentage production, percentage first class eggs and percentage second class eggs) and water consumption showed no substantial difference between phases. Mean daily mortality saw a decrease between the pre-Treatment (2.43 birds per day) to the Treatment phase (0.50 birds per day) which remained into the post-Treatment phase (0.53 birds per day). This decrease in mortality was due to a singular smothering event during the pre-Treatment phase.

Mean daily bird weight and feed consumption per bird increased between phases, in-line with breed standards (ISA, 2021). Feed consumption increased from 127 grams per bird in the pre-Treatment phase to 133 grams per bird during the Treatment phase and 132 grams per bird in the post-Treatment phase. Breed standards predict an increase on average of 2 grams per phase given the age increase throughout the trial (ISA, 2021). Therefore, while an increase is expected between the pre-Treatment and Treatment phase, the larger increase could be due to change in palatability of the feed from the additive, but this is not supported by the consumption plateau seen upon removal of the additive.

Overall, our study provides novel data demonstrating the potential for passionflower-containing supplements to be beneficial in reducing feather pecking behavior in laying hens, though we acknowledge the limitations posed by a small case study. Considering the billions of laying hens kept worldwide and the scale of negative welfare and economic consequences linked to feather pecking, we advocate for further research to explore the mechanism and longer-term effects of passionflower.

## Declaration of competing interests

The authors declare the following financial interests/personal relationships which may be considered as potential competing interests: Helen Gray reports financial support was provided by Techna UK. Elizabeth Brass reports financial support was provided by Techna UK. Coauthor conducted an undergraduate internship with Techna UK Ltd (EB). Techna UK supplied the product Gallicalm free of charge but had no role in experimental design, analysis or interpretation of results. If there are other authors, they declare that they have no known competing financial interests or personal relationships that could have appeared to influence the work reported in this paper.
